# AnArtomy: Arts, Anatomy and Medicine - Human Beings Being Human

**DOI:** 10.15694/mep.2018.0000204.1

**Published:** 2018-09-10

**Authors:** Ian Walsh, Joseph Quinn, Andrea Spencer, Helen Noble

**Affiliations:** 1 Queen's University Belfast

**Keywords:** Art in Health, Medical Humanities, Art and Anatomy

## Abstract

This article was migrated. The article was marked as recommended.

Background

The study of anatomy underpins medical education and is an important facet of clinical practice in various diverse disciplines. We explored the dynamic relationship between arts, anatomy and medicine, along the continuum axis of anatomy, medicine, healthcare and art.

Aims

1. to foster and gauge artistic and creative expression within the context of medical science and practice.

2. to generate representative artwork examining the relationship between art, medicine and healthcare.

Methods

Two purposefully open and expressive creative workshops were held within the cadaveric dissection laboratory of the Queen’s University Department of Anatomy; with awide variety of artistic substrates available for faculty and student participants. Themes included: the relationship between art and medicine, the impact art and science have upon each other and the effects of creativity on wellbeing. Accompanying questionnaires included a quantification of perceived relationships between art and medicine; with an estimation of connectedness to feelings. Qualitative items within each questionnaire also addressed key humanistic questions. Comparative analysis of quantitative results was by Student’s t-testing; statistical significance being p values <0.05.

Results, Summary and Conclusions

There was a statistically significant increase in “connectedness to feelings” amongst participants over the course of the workshop. There was a trend for participants to agree or strongly agree that art and medicine were important to each other. Qualitative responses changed from specific, task-oriented hopes to responses more aligned with social/gregarious themes and those related to higher order functioning. Humanistic responses changed across the entire group from a largely fixed inclusion of the concept of emotions to broader, more altruistic visions; inclusive of communal, social views. There was a noticeable shift in emphasis from succinctly defined descriptive terms to more expressive terms; reflective and inclusive of caring, holistic practice. The most arresting and compelling results were those of the resulting representative artwork.

## Introduction

***“Medicine is not only a science; it is also an art. It does not consist of compounding pills and plasters; it deals with the very processes of life, which must be understood before they may be guided” - Paracelsus***

***“Wherever the art of medicine is loved, there is also a love of humanity”-Hippocrates***

Anatomy is a science, medicine less so; healthcare even less again.

The knowledge and principles of anatomy as a grounding for clinical learning in healthcare cannot be overstated ( Sbayeh *et al,*2016) The study of anatomy underpins medical education and is a key central feature of the practice of surgery and radiology, as well as forming an important facet of clinical practice in a diversity of disciplines such as internal medicine, anaesthetics and neurology; to name but a few specialties.

Much of anatomical learning is based around imagery and the rendering of representative detail, which often leads students and practitioners of this science into the creative realm of artistic portrayal. The teaching of anatomy is underpinned by the work of key individuals, such as Frank Netter (1906 - 1991); often referred to as “The DaVinci of Anatomy”. Netter was a surgeon and medical illustrator, who produced circa 400 illustrations, which featured in key anatomical texts. His work was compared to and superseded by the epochal illustration of Andreas Vesalius (1514 - 1564) and Leonardo DaVinci (1452 - 1590). Vesalius was a surgeon, anatomist, artist and scientist, whose artistic output was both voluminous and hugely influential. Da Vinci’s artistic background informed his illustrations of cadaveric dissection, imbuing these with dynamics and life. It is in the interface between his inhabited worlds of art and science that his phenomenal creative output proves most fascinating. Later, the illustrated works of Henry Gray (1821 - 1865), an anatomist and surgeon became a central feature of anatomical and surgical texts, including his own seminal publications *Anatomy of the Human Body* in 1918 [more commonly know as *Gray’s Anatomy*] ( Gray, 1859).

Medicine deals with the human condition, often at it’s most fragile, dealing with the wide range of the human condition; encompassing suffering, illness and death or recovery. This spectrum of the human condition contains the central substrate for the production of all forms of art ( Walsh 2017). The dynamic and mutually beneficial relationship between art and medicine has been increasingly recognised and explored, particularly over the last several years. Cole et al (2015) believe that arts and humanities can contribute to “ *cultivating personality, intellectual curiosity, emotional honesty, social awareness and the exercise of sound judgement and moral imagination - virtues and skills indispensable to good doctoring* ( Cole et al, 2015)16. The ability and potential of art to comfort, heal, and help individuals find hope in illness and suffering has become recognized as a role for art in healthcare ( Hanna, Rollins and Lewis, 2017).

It was with this dynamic relationship between arts, anatomy and medicine in mind that we embarked upon this project; recognising and exploring the continuum axis of anatomy, medicine, healthcare and art.

## Methods

An open invitation was sent to all first and second year undergraduates at Queen’s University Belfast during the Development Weeks part of their study program (this is a typically fallow period between the commencement of main curricular studies, wherein students and teaching faculty are encouraged to volunteer for additional teaching and learning activity to broaden their academic experience at the university). Students registered online for 2 consecutive daily workshops, which were designed by the Artist in Residence ( *AS*) at the Department of Anatomy.

All creative activity took place within the cadaveric dissection laboratory of the Queen’s University Department of Anatomy. All participants were briefed at the outset on the principles, regulations and legislation relating to the Human Tissue Authority, as pertinent to the venue.

The workshop design was purposefully open and expressive, with a wide variety of artistic substrates being made available for both faculty and student participants. In addition to resources such as drawing materials including paint, pencil, crayon and ink printing blocks, all participants were given free access to a wide range of additional materials with the potential for artistic deployment. These materials included substrates such as willow, wire, foliage, plumage, cardboard and aluminium.

At the start of the first day of the workshops, the lead artist demonstrated techniques for working with the materials supplied, including demonstrations in the safe deployment of wire cutting tools, ink printing and methods for entwining willow, together with sculpting techniques.

Participants were encouraged to work in groups and discuss their direction of creative travel before engaging with the materials. It was explained to all involved that the first two hours should be spent in acclimatising and growing accustomed to the various resources and how best to interact with them. Faculty freely circulated amongst participants, answering questions and providing anecdotal narratives to inform artistic construction. Themes raised included:

• the relationship between art and medicine
• the impact art and science have upon each other
• the effects of creativity on wellbeing

All participants were asked to complete a questionnaire ( Appendix 1) at the start of the day one workshop and a separate questionnaire at the end of day two. These questionnaires were designed to gather information on participant demographics, current academic status and previous experience with artistic endeavour, together with Likert scale quantification of participant’s perceived relationships between art and medicine; with an estimation of connectedness to their own feelings. The key qualitative questions, as free text responses included in the questionnaires were:

• What are you hoping to personally gain from this workshop?
• What do you predict or hope will be the result(s) of the workshop?
• What does *“human being”* mean to you?
• What does *“being human”*mean to you?
• What does *“being a doctor”*mean to you?

Quantitative questions and statements (Likert Scales) were based on three key statements:

• Art is important to medicine
• Medicine is important to art
• How connected to your feelings do you feel right now?

### Data analysis

Likert scale responses were coded and exported to standard (Excel) spreadsheet format, wherein descriptive and comparative statistical analysis was undertaken. Comparative analysis was by Student’s t-testing, with statistical significance taken at p values <0.05).

A variety of artwork forms were created by the participants, largely working in teams which formed and developed throughout the workshops. Accompanying explanatory commentary and narrative was encouraged in the production of this artwork, to delineate the genesis of each piece. Such commentary was guided toward the above themes, as they emerged and evolved.

Digital photographic representation of artistic activity in progress and upon completion of all creative artefact was undertaken throughout the workshops, deploying designated digital photographic equipment designated to the Department of Anatomy in line with Human Tissue Authority regulations and legislation.

## Results/Analysis

The results have been divided into:

(1) Demographic results

(2) Qualitative results derived from questionnaire free text responses and direct verbal comment during workshop activity.

(3) Quantitative results - derived from analysis of Likert Scale responses to the three key statements.

(4) The multifarious artworks produced by participants. Representative explanatory commentaries were aligned with each piece to provide context and narrative.

### 1. Demographics

Mean participant age was 25y (range 19-55). The majority (21/28; 75%) of participants were students, mostly (17/21; 81%) undergraduates. Students hailed from subject areas such as Medicine, Biomedical Science, Astrophysics, English and Law; the predominant representation being from biological/biomedical sciences.

(10/21; 48%) students and (3/7; 42%) faculty reported previous experience with art; ranging from studying art at school, through involvement with craft works, mural painting and advanced musicianship.

### 2. Qualitative Results (questionnaire responses)

Exemplar questionnaire responses are represented in Table 1 (Appendix), with a delineation of these between those immediately before the workshop began and those at the end of all creative activity:

### 3. Quantitative Results

**Figure 1.**
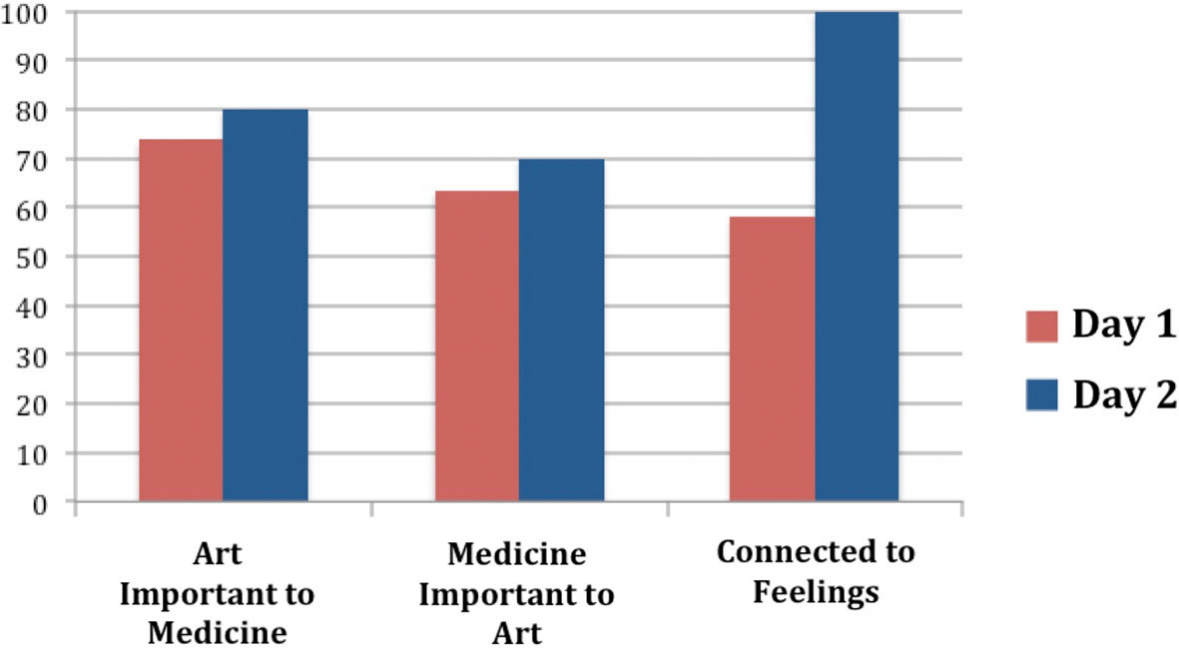
Responses to Key Statements:

Analysis of the Likert Scale responses to the key statements are illustrated in Figure 1.

There was a statistically insignificant trend for participants to agree or strongly agree that art is important to medicine (p = 0.4) and that medicine is important to art (p = 0.3). In contrast, there was a statistically significant (p = 0.03) increase in “connectedness to feelings” amongst participants over the course of the two day workshop.

### 4. Artistic Artefact

The resultant artefacts took the form of sculpture, often accompanied by contextual narratives; the latter of which provided rich descriptions of each artwork’s genesis, evolution, construction and creation. These narratives and the artworks themselves also included and provided significant personal back stories from the participants; as each creative exercise allowed back stories and context to emerge. Sharing of experiences during such creative activity was an invaluable aspect of the entire activity and helped forge both collaborative efforts and subsequent friendships, as alluded to in Table 1.

A selection of the creative works are shown in Illustrations 1-6.

Illustrations 1 and 2 show representations of the human heart and lungs, fashioned from tape, plastic conduit tubing, surgical gauze and plaster of Paris. The structures are visually arresting, especially with the strikingly vivid representation of blood and blood vessels in “Heart”, which are roughly representative of the vessels of the adult coronary artery circulation. These artworks, created by medical students, are both somehow anatomically correct, yet emotive.

**Illustration 1.**
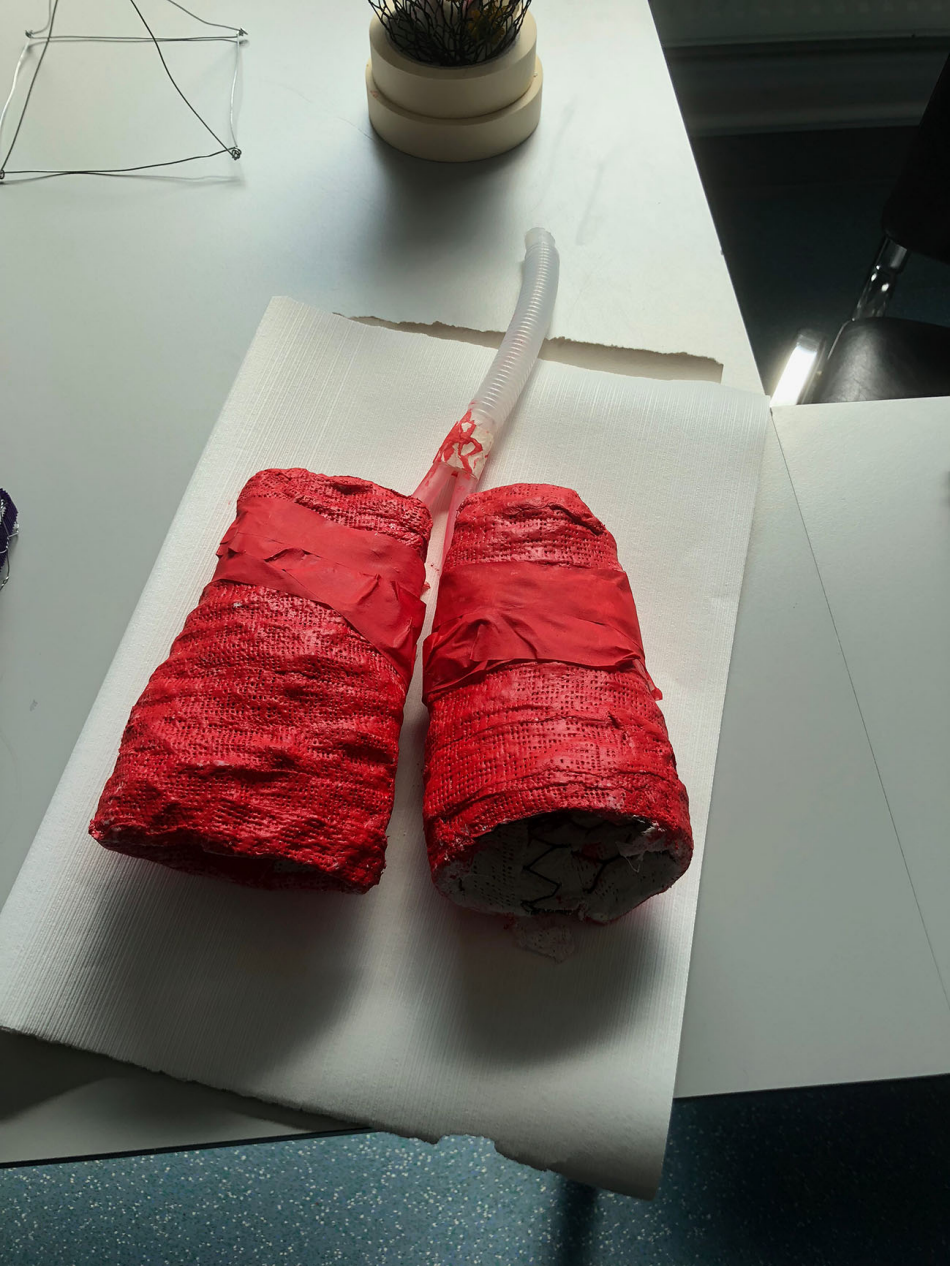
“lungs”:

**Illustration 2.**
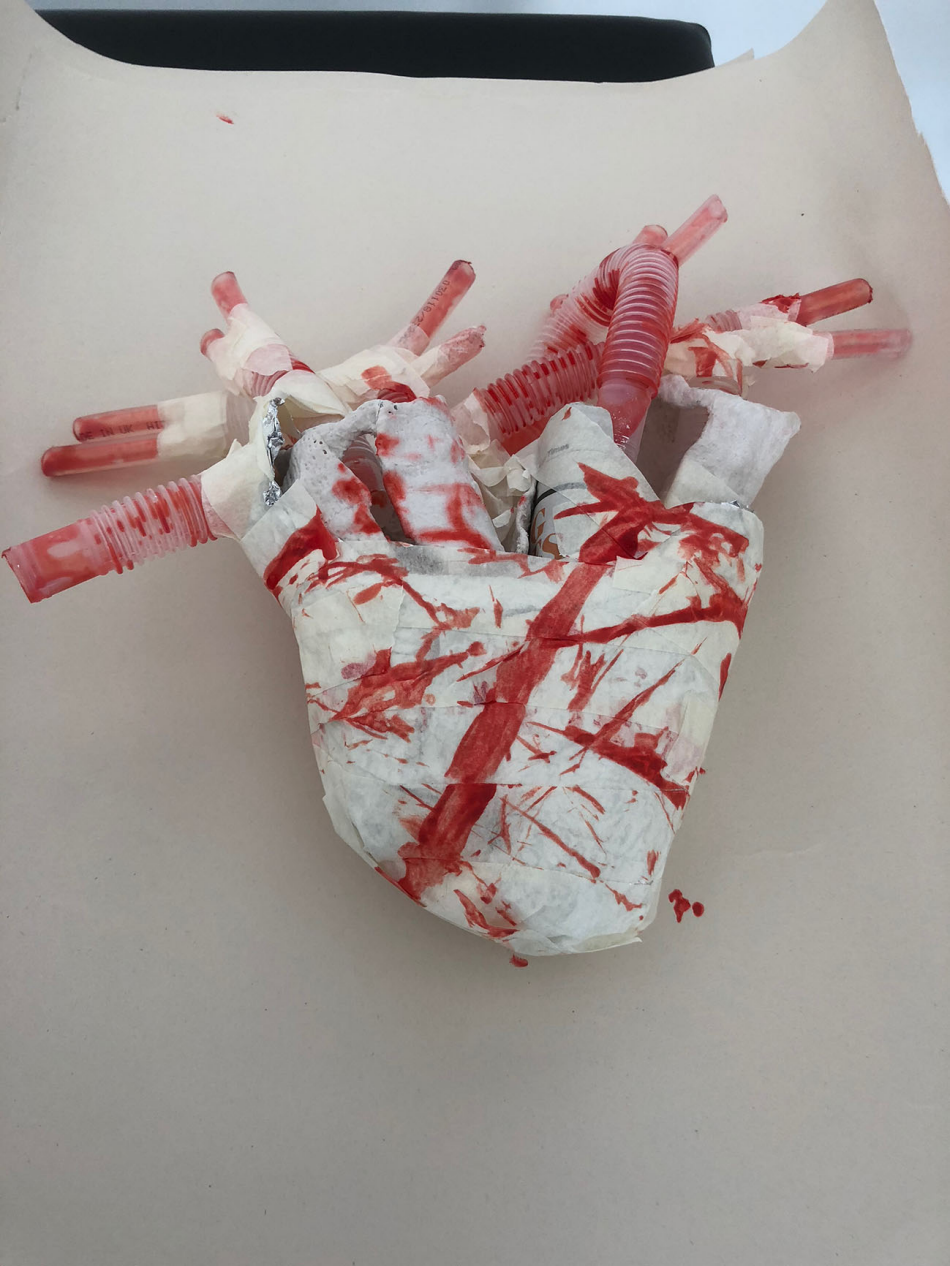
“Heart”:

Illustration 3 (The Body and Nature) is arresting in its’imagery, combining substrate from nature (foliage) with typically inert material (wire) and medical matter (gypsum; “plaster of Paris”). Again a representative heart, replete with great vessels coronary arterial supply entwines and contains a deeply olive green branch and still-living leaves. The inscription could be considered a subtext for the entire project: *“exploring anatomical connections abstractly; linking with natural forms”.*

**Illustration 3.**
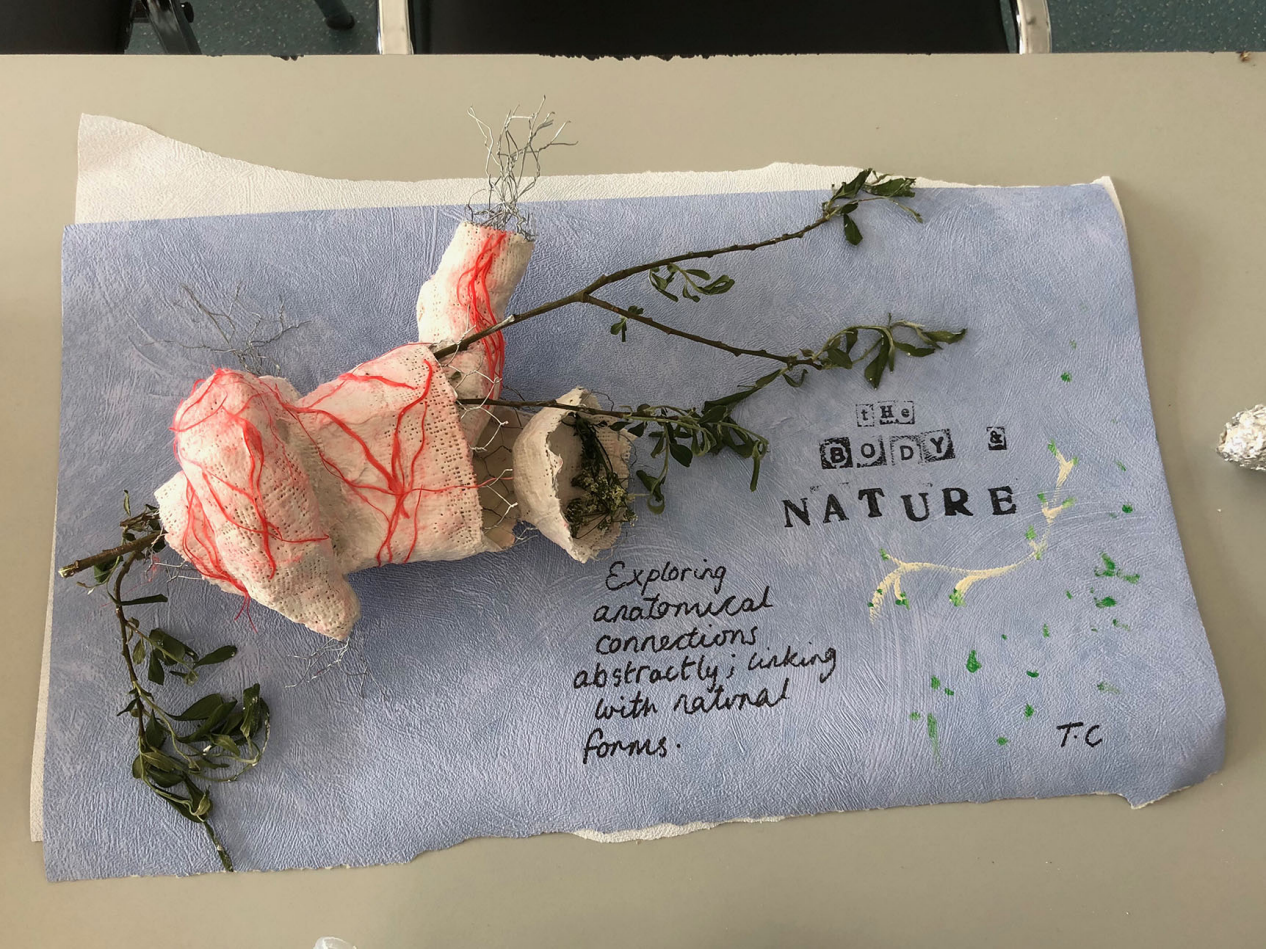
“The Body & Nature”:

“Leg Within a Leg” (see Illustrations 4 and 5) encompasses and expands a personal narrative. The medical student sculptor explained that her contribution was inspired by the story of her brother, who had undergone amputative surgery of his lower limb as part of the treatment for his osteosarcoma. The student was privy to information relating to prosthestics in light of this personal connection, together with her knowledge accrued during her anatomical and medical studies. Through the creation of this striking sculpture of wire mesh and gypsum, she was able to articulate feelings relating to her sibling’s disarticulation, simultaneously expanding on the technical challenges of prosthetic design.

**Illustration 4.**
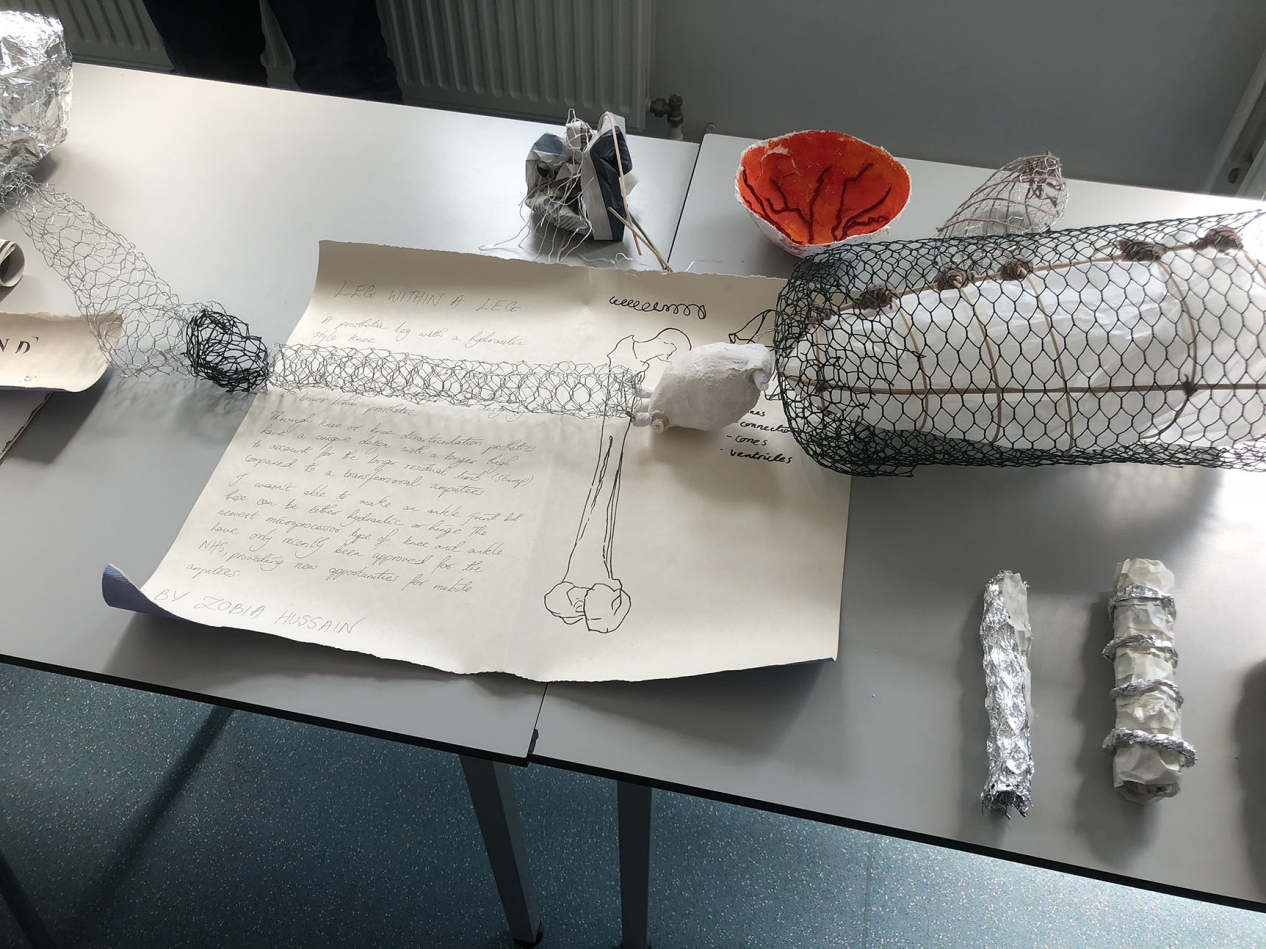
“Leg Within a Leg”:

**Illustration 5.**
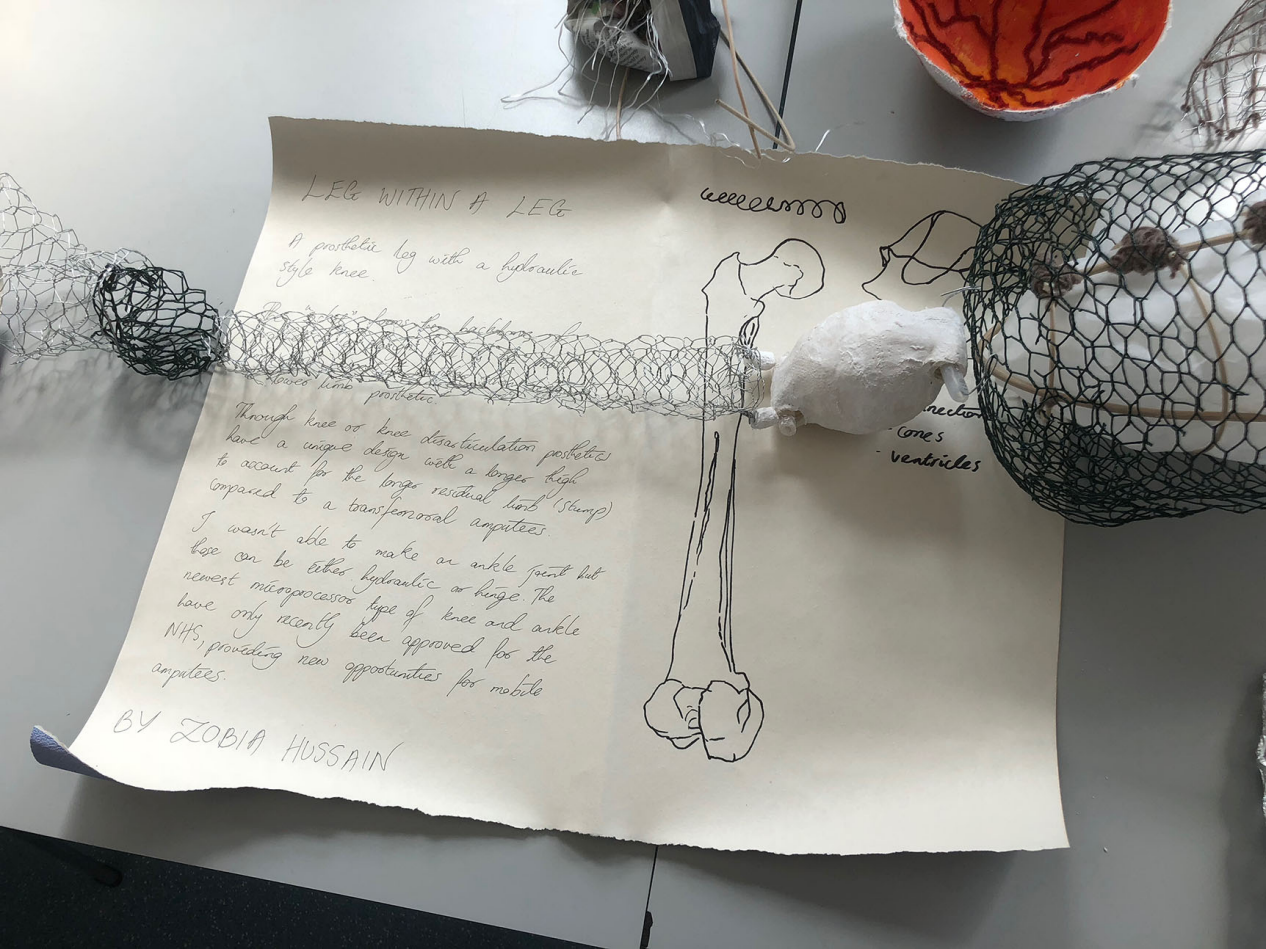
“Leg Within a Leg” (close-up view of narrative):

“Flower Lungs” (Illustration 6) represents an innovative use of willow, surgical tape, foliage and various brightly coloured fabrics, caged within wire mesh. The resultant sculpture is a representation of the adult human trachea (together with cartilaginous [willow] rings), bronchi and lungs. The concept of lower airway branching is represented by actual branches of verdure and the “floral” lung substance somehow suggests the breath of life.

**Illustration 6.**
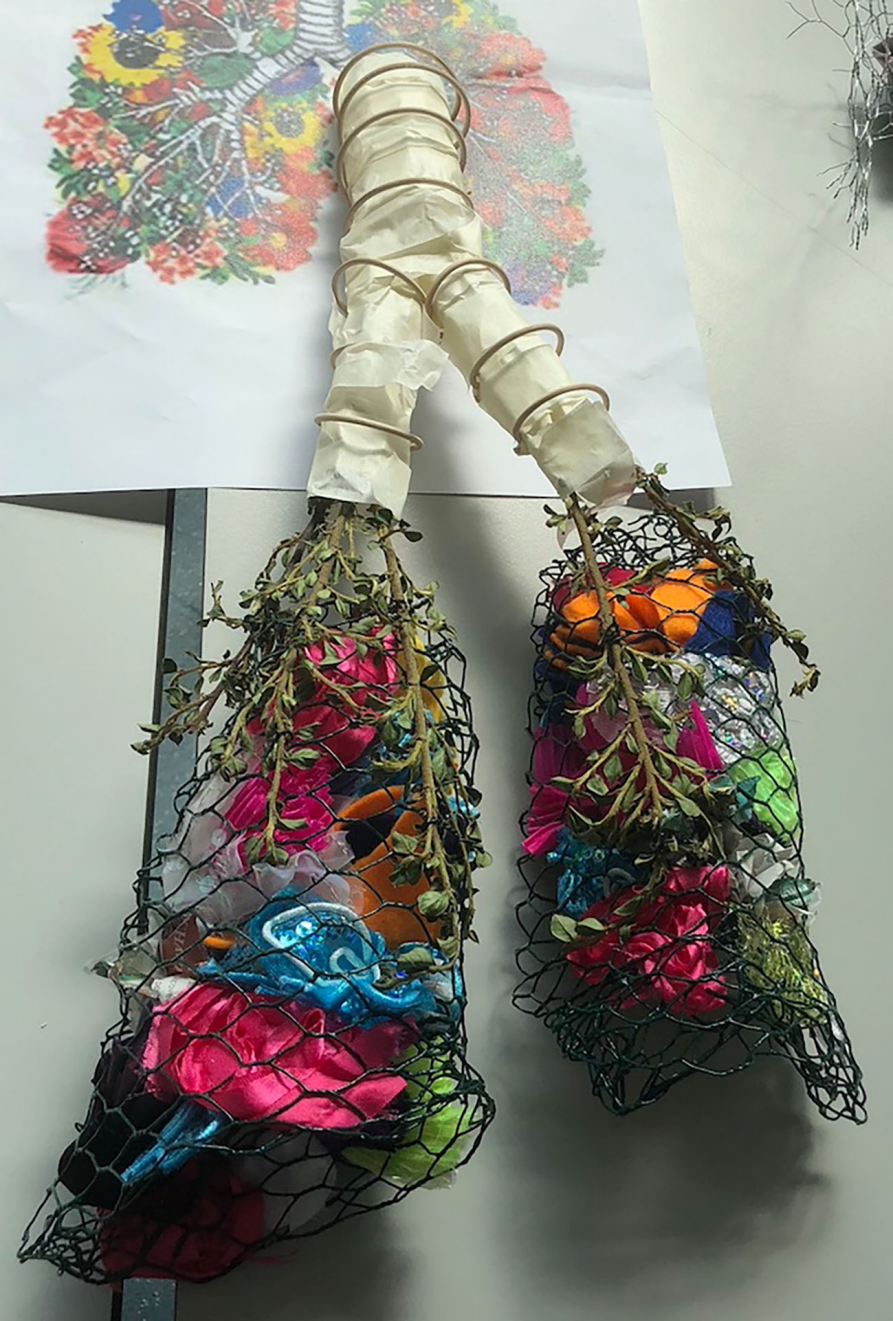
“Flower Lungs”:

## Discussion

Medicine can at any one time be referred to as a discipline, a profession, a subject, a field, a specialty, a career or a practice ( Walsh, 2017). The practice of medicine is deployed within the arena of healthcare provision, wherein it is now recognized that human factors play a hugely influential role, both in terms of efficacy, efficiency and safety. Such human factors include technical skills, behavioural skills and encompass human-human interactions, together with ergonomics (human-machine and human-environment) interactions. Humans are the central feature of medicine and healthcare; in the form of professionals, patients, relatives and carers. Effective and compassionate healthcare should embrace and incorporate this tenet, always being mindful that medicine is not wholly a science, nor entirely an art; but occupies a variable position on an axis betwixt the two.The oft-quoted “doctor-patient” relationship is essentially a humanistic construct and is considered a central pillar of clinical practice. Within healthcare provision, particularly in the welcome era of multidisciplinary practice, the relationship between healthcare professionals is, by necessity and definition, also enterprisingly humanistic.

Art-mediated peer learning has been shown to enhance both an appreciation of both the value of the doctor-patient relationship and holistic care, with the potential to educate on the humanistic aspects of clinical practice ( Potash and Chen, 2014). Our workshop facilitated human-human interaction and human-material interaction in an open, safe and supportive environment which was truly interdisciplinary; with faculty representation from domains such as visual art, music, surgery, anatomy, physiology. Likewise, the student participants hailed from diverse fields such as medicine, law, biomedical and biological science, as well as astrophysics and engineering. The qualitative feedback results indicated that there evolved a sense of teamwork, camaraderie and friendship - all aspirational qualities of effective and caring healthcare teams. The participant groupings which developed were variform in terms of: (1) vertical integration; there was distinct representation across the undergraduate-postgraduate-faculty continuum and (2) interdisciplinarity; horizontal integration of participants occurred effortlessly amongst subjects and disciplines as diverse as physics, law, biomedical sciences and english.

### Qualitative Results

#### Questionnaire Responses

The preliminary items on the questionnaires were directed towards the participant’s aspirations for the workshops. It can be seen from the responses before and after the workshop, that these aspirations distinctly changed from specific, task-oriented hopes (e.g. *“insight into anatomy and medicine in creative form”*; *“learn more about the human body and how art relates to the body”*; *“improve my manual dexterity”*; *“learn how to do more 3D work”*) to responses more aligned with social and gregarious themes, as well as those related to higher order functioning (e.g. *“friends, meeting new people, having fun”*; *“people have a good time, teamwork established and new friends are made”*; *“a closer relationship to my lecturers”*; *“inspiration and insight”*; *“build creativity”*;). It is encouraging to note such a change, especially as such would be considered as conducive to communal and supportive learning.

Subsequent questionnaire items allowed for exploration and examination of views on humanistic concepts and the relationships betwixt arts and healthcare. Views of what it means to be human and what human beings are defined by varied amongst participants, dependent upon their academic background. When asked to define “human being”, there was no noticeable difference in responses at the beginning and end of the workshop. Those participants with a greater scientific background weighting tended to be provide responses associated with positivist language (e.g. *“higher organism”; “living creature”; “a species; something that can be studied”*), whereas those with a less scientific background tended to respond with constructivist/creative language (e.g. *“being alive”; “ability to think”*; *“living”*; *“soul, love”*). However, when asked about “being human”, responses changed across the entire group from a largely fixed inclusion of the concept of emotions ( *Being alive, having consciousness, emotions, feelings”*; *“having emotions”; “being emotional, achieving things, thinking, feeling...”*) to broader, more altruistic visions ( *“being part of something more”; “how you live your life”*; *“having a life worth living”*). The latter responses were again more inclusive of a communal, social view ( *“experience new things and build relationship”*; “ *our human nature*). Again, this is a welcome observation and one which bodes well for interdisciplinary learning.

When asked about what ‘being a doctor” meant; as the artistic collaborative activity progressed, there was a noticeable shift in emphasis from succinctly defined descriptive terms ( *“fulfilling the requirements/principles of what it means to be a doctor”*, *“being safe and hopefully knowledgeable”*, *“someone who has a degree in medicine”*) to terms which were more expressive, reflective and inclusive of caring, holistic practice ( *“to save people”*, *“having empathy, being a listener and carer to those in need”*, *“curing the body or aiding it in death - providing comfort”*).

It may well be that engagement with, and immersion in, artistically creative activity fostered these changes in perspective; allowing broader thinking to emerge when considering key features of the human condition. A similar process may have underpinned the shift in perspective of participants when considering the cardinal features of a doctor, as it was apparent that a greater consideration of higher order functioning such as empathy, caring and healing had come to the fore. An additional factor in such shifts may have been the influence of collaborative working. Free conversation and open thinking was encouraged amongst participants from disparate backgrounds; perhaps allowing for a greater understanding of healthcare professional roles as historically typical interdisciplinary barriers lowered organically.

### Quantitative Results

Figure 1. shows that, as the creative collaborations developed, there was a statistically significant increase in the extent to which participants felt connected to their feelings.

In essence, this was an unstated aim of the project; as we negotiated the complex pathway between science and art, there was somehow a tacit agreement amongst all involved that we were exploring something connected to both feelings and higher order functioning. This was expressed in some of the questionnaire responses discussed above (c.f. *“contemplating surroundings at a higher level”*, *“having thoughts, feelings and emotions”*) and such responses may have indicated an evolving awareness of feelings as a central feature of both the workshop. It was hoped that the questionnaires themselves would prompt reflection, discussion and an appreciation of the emotional landscape. This may explain the genesis of the encouraging and significant (including statistically) results. The increased identification with feelings may also have been augmented by the collaborative nature of the creative activity, wherein free conversation and discussion of topics such as feeling and emotion were fostered and encouraged.

### Artistic Artefact

For all involved, the most arresting and compelling results were those of the resulting representative artwork. The nature of the production and creation of this work was entirely organic. Themes emerged within groups and minimal direction was required from faculty to progress any of the work. There was an initial hesitancy at the outset of the creative activity, as participants grew accustomed to the variform artistic substrates and materials. However, once the basic material handling skills were explained and demonstrated to the participants by the artistic director ( *AS*), creative activity quickly got underway. Each completed work was largely the result of group activity, especially amongst the student body. Faculty participants tended to work solo, although free conversation and collaborative comment was clearly evident as work progressed. It is perhaps this collaborative facet of the project which proved most fruitful. It is difficult to envision that such arresting artwork could have resulted from efforts in a non-networked environment. On the subject of environment, this workshop was particularly unique by it’s setting within a cadaveric dissection laboratory. Such environs would not be immediately clearly evidenced by the resultant artwork. It is hoped that this unconventional setting itself provided food for thought and consideration as collaborative creation progressed. This is discussed in the final paragraph.

Placing the scientific and medical alongside the creative allows for a uniquely fertile soil. Participants may come to realisations hitherto unknown or unrecognised; whereby the similarities and common ground of the art of medicine and the science of creativity may come to light. This is the arena inhabited by the polymath giants such as Vesalius, Da Vinci, Gray and Netter, as described in the introductory section. We replicated such a propagative environment on a microcosmic scale and contend that the results were fruitful in terms of supportive creativity and producing representative artistic content of worth.

Several authors have commented on the potential for engagement with arts and humanities to enhance imagination and empathy in healthcare, as well as linking emotional and rational brain functions ( Katz and Khoshbin, 2014; Riess, 2010). It has also been demonstrated that facilitating undergraduate medical students to examine fine art allows them to become more observant, self-caring and empathetic ( Hojat *et al*, 2009).

Within the critical realm of patient safety in healthcare, it has been argued that immersion in arts-based activity may enhance self-awareness and recognition of the impact of emotions on clinical judgement ( Potash *et al* 2014).

Art can also effect culture change, especially around health provision issues such as literacy, understanding and trust, thus affording opportunities for prevention and cure ( Bonder & Martin, 2013; Govindasamy *et al*, 2014; Sonke & Lee, 2016). A more humanistic approach to the practice of healthcare has become increasingly evident, relevant and welcome in recent years and holistic care has now been recognised as the aspirational standard for modern healthcare provision in the modern ( Serlin, 2007) Within this holistic paradigm, the arts can uniquely provide an environment and opportunity to create environments conducive to recovery, healing and health for both patients and carers alike (Hanna *et al*, 2017).

In relation to medical learning within a cadaveric dissection setting, George Dickinson eloquently opined” *Medical training is more accurately characterized as a process of socialization, that medical students are not only “passing through” an experience intended to refine previously established values, but that those previous identities must be repudiated and new and countervailing identities assumed...*” ( Dickinson *et al*, 1997). We have thus explored this arena specifically within the unique setting of an anatomy dissection laboratory; an environment typically and traditionally associated with sterility and the scientific approach, conjuring up stark, dispassionate imagery. The results of this project; particularly the life-imbued artefacts which were produced, have demonstrated that such a setting need not necessarily be stagnating, but can be creatively invigorating. Such environs are necessarily associated with the human condition, particularly dying and death. We argue that this association may render such spaces as distinctive places for learning, collaboration and creativity.

## Conclusion

The full spectrum of the human condition is encountered in healthcare, which thus contains the central substrate for the production of art. Fostering artistic and creative expression within the context of medical science and practice allows for a holistic approach to science and health, whilst similarly providing a uniquely humanistic climate for artistic creation. Exploring stark, singular spaces such as the cadaveric dissection laboratory we housed this study in allows for rich, experiential, creative learning and attitudinal change.

## Take Home Messages

• Art and medicine are uniquely placed as overlapping, potentially synergistic disciplines
• Artistic and creative expression within the context of medical science fosters a holistic approach
• Medical science allows access to key features of the human condition, which is the central substrate for art
• Typically stark scientific surroundings such as anatomy dissection laboratories can provide uniquely inspirational and creative learning environments

## Notes On Contributors

Mr Ian K Walsh is a Clinical Academic and Urological Surgeon at Queen’s University Belfast, Belfast Trust and Kingsbridge Hospital. He is Chair of the Northern Ireland Healthcare Human Factors and Transatlantic Arts, Humanities and Health groups.

Dr Joseph Quinn is a lecturer in anatomy at Queen’s University Belfast, who teaches anatomy, physiology and neuroanatomy. He champions scholarly support and leads student development. He has published widely in international peer-reviewed journals; ranging from neurosciences to medicinal chemistry.

Andrea Spencer is Residential Artist at the Department of Anatomy and active member of The Renal Arts Group at Queen’s University Belfast. Her prolific body of work references the human condition, expressed through teh poetics of glass; utilising a visual language common to both science adn art.

Dr Helen Noble is a Senior Lecturer at Queen’s University Belfast, where she chairs the Renal Arts group. Her research explores creative activity in palliative settings and she leads the Mindfulness and Resilience support and research group.

## Appendices

**Table 1.**

Question	Responses at start of workshop	Responses at end of workshop
**What are you personally hoping to gain from this workshop?**	*A better understanding of anatomy* (medical student) *Creativity and how art and medicine can relate* (biomedical student) *To be able to see in which way medicine and art can overlap and how this can have an impact on how we are as doctors* (medical student) *More experience with art* (biomedical student) *Insight into anatomy and medicine in creative form* (postgraduate physics student) *Learn more about the human body and how art relates to the body* (english student) *Improve my art skills and better understanding of anatomy* (medical student) *To explore medicine in an artistic view* (medical student) *Just have fun and learn about anatomy through art* (medical student) *Having an art outlet and getting to make something to have in the office* (faculty) *Having a bit of fun messing around with some arty stuff* (medical student) *Fun! Sculpture skills* (medical student) *Fun. See anatomy in a new way* (medical student) *Make some nice stuff, meet new people* (biological sciences student) *Improve my manual dexterity* (medical student) *New experience - tap into creativity that may be latent - have fun* (faculty)	*Friends, meeting new people, having fun* (biomedical student) *Improve art skills and understanding of anatomy* (medical student) *Do something I love* (medical student) *Practical skills, friendship* (biomedical student) *Inspiration and insight* (physics student) *Art skills, learning more about a certain body part* (law student) *Build creativity* (medical student) *A closer relationship to my lecturers friendship* (medical student) *Learn how to build an anatomy structure and understand it* (biological sciences student)
**What do you predict or hope will be the result(s) of the workshop?**	*A better understanding of anatomy* (medical student) *Final pieces/projects/sculptures* (biomedical student) *Being able to prove that art and medicine are not mutually exclusive* (medical student) *To understand the connection between art and medicine* (medical student) *Create a piece of art* (english student) *An impressive anatomical model* (biological sciences student) *Improved artistic skill* (medical student) *Learn more about anatomical specimens* (medical student) *Learn how to make models* (law student) *Collaboration with staff and students. Staff wellbeing. Introduce non-healthcare students to anatomy* (faculty) *Making a sculpture* (medical student) *Make new friends* (biological sciences student) *Meet new people* (english student) *Amazing experience* (english student) *Learn how to do more 3D work* (medical student) *Create something new. See if I’m inspired* (faculty)	*People have a good time, teamwork established and new friends are made* (medical student) *Fun!* (medical student) *I have made new friends and become closer with others* (biomedical student) *Have a resulting artwork* (english student) *Meet new people and build communication*(biomedical student) *A new perspective on art and medicine* (medical student) *Have more interest in biology* (physics student)
***What does “human being” mean to you?***	*Being alive* (biomedical student) *The basic classification of who we are as individuals* (medical student) *Anatomical. Any person with thoughts, emotions, moral compass* (biomedical student) *Living organisms that have an awareness of their own conscience and can communicate on a higher level*(postgraduate physics student) *Human body* (english student) *A person* (biomedical student) *A soul encased in a beautiful biological machine* (medical student) *A living creature of the human race* (biomedical student) *A species; something that can be studied* (faculty) *Physical human body and all it’s parts* (medical student) *A member of the human race* (medical student) *Being a higher organism with a soul* (biological sciences student) *Animals with the ability to think* (law student) *A person* (medical student) *Soul. Love. Forge connections. Relationships* (faculty)	*A person who is living* (biological sciences student) *Being a person; our species* (medical student) *A living organism that has articulated speech and communication, ability to have advanced emotions* (physics student) *Being alive and having a human body* (biomedical sciences student) *A high level organism*(medical student) *Being alive; having thoughts; breathing* (law student) *A living thing* (english student)
***What does “being human” mean to you?***	*Being happy* (medical student) *Being part of the human race* (biomedical student) *Exhibiting human emotional qualities* (medical student) *Having compassion, emotions* (medical student) *To be an active, thoughtful mind and body that can think of the deeper meanings of life* (astrophysics student) *Having emotions*(biological sciences student) *Being compassionate*(medical student) *Having thoughts, feelings and emotions* (medical student) *Being emotional, achieving things, thinking, feeling, exploring, failing, discovering* (faculty) *Being alive, having consciousness, emotions, feelings, etc.* (medical student) *Being kind and compassionate Experiencing a range of emotions* (medical student) *Having a life and feelings* (biological sciences student) *Being myself and having integrity* (law student) *Have a life of meaning* (english student) *Trying - making mistakes and failing, but failing better* (faculty)	*Having emotions and feelings that differentiate from other beings* (medical student) *Feeling emotions*(biological sciences student) *Our human nature* (medical student) *Contemplating surroundings at a higher level. Living with ambition and awareness of future.*(astrophysics student) *Having a life worth living* (biomedical sciences student) *Be able to experience new things and build relationships*(medical student) *Being part of something more*(medical student) *Being self aware. Bones and blood.* (law student) *How you live your life* (english student)
***What does “being a doctor” mean to you?***	*Saving lives* (medical student) *Being able to improve others’ lives* (biomedical student) *Fulfilling the requirements/principles of what it means to be a doctor* (medical student) *Healer, protector, kind, guide, leader* (medical student) *To understand scientifically, the body and mind to help every individual under your care* (astrophysics student) *Long working hours, helping patients, connecting with people* (english student) *Someone who has a degree in medicine* (biological sciences student) *To help others with my skill* (medical student) *Having advanced studies in certain subjects* (medical student) *Helping people who are sick and injured* (law student) *Being safe and hopefully knowledgeable. Very stressful, but also very rewarding* (faculty) *Practicing medicine, looking after the physical and mental wellbeing of others.* (medical student) *Practicing medicine*(medical student) *Healing human, earning money, stressful job* (biological sciences student) *Help others with illness, stressful* (law student) *High earner, help people in need mentally and physically* (english student) *Lots of money, stress* (english student) *Being a patient’s advocate for whatever needs they may have, act as someone to rely on in times of vulnerability* (medical student) *Skills centred but also empathy and human based - equally important but disregarded* (faculty)	*Having empathy, being a listener and carer to those in need*(medical student) *Medical degree, someone who improves quality of life* (biological sciences student) *Curing the body or aiding it in death - providing comfort*(medical student) *Healer, carer, knowledge of the body*(astrophysics student) *A human, long hours, hard work, helping patients in need*(biomedical sciences student) *Help heal people mentally and physically*(medical student) *Being a healer* (medical student) *Fixing humans* (law student) *To save people*(english student)
