# Supplementary material for: AnArtomy: Arts, Anatomy and Medicine - Human Beings Being Human
Source: MedEdPublish (2016). 2018 Sep 10;7:204. [Version 1] doi: 10.15694/mep.2018.0000204.1 (PMC10701823; doi:10.15694/mep.2018.0000204.1)
Supplement: Peer review history

# Reviewer report: Reviewer response for version 1

**DOI:** 10.21956/mep.19646.r29289

**Published:** 2019-06-28

**Authors:** Iain Keenan

**Affiliations:** 1 Newcastle University

This review has been migrated. The reviewer awarded 3 stars out of 5 Many thanks to the authors for this interesting article. Advocating the value of art-based learning approaches in anatomy is certainly important, based on the theory and evidence supporting how such approaches can be effective in anatomy education. I would liked to have read more about this within a more detailed introduction. Given the subject matter, it may have been effective to have approached this work from the interpretivist paradigm. The stance appears not be coherent however, as it contains both post-positivist (Likert-type items with statistical analysis) and interpretivist (free-text items with qualitative analysis). Quantitative thematic analysis of the free-text data would provide coherence with a post-positivist stance. A description of how the thematic analysis was performed would also be welcome. These data should also be presented in the results section rather than the discussion. The statistical data would benefit from a more visually appealing figure, from the inclusion of standard deviation error bars in the chart and also from p values indicated as p > 0.05 (NS) or p The authors may find our 12 tips paper of interest: https://www.mededpublish.org/manuscripts/1012

Reviewer Expertise:

NA

No decision status is available

# Reviewer report: Reviewer response for version 1

**DOI:** 10.21956/mep.19646.r29288

**Published:** 2018-09-10

**Authors:** Trevor Gibbs

**Affiliations:** 1 AMEE

This review has been migrated. The reviewer awarded 3 stars out of 5 An interesting paper to read, although I am a little unsure what this really adds to our thinking about how the humanities can influence our future thinking about educating our new students and how the humanities fit into our new curricula. I was very impressed by the "sculptures" created by the students and would very much agree that there was a clear development of team learning and an expression of the students inner-self and their ability to look at the human through rather tangential eyes - I think the paper could have been improved by allowing a more discussive process, post making the sculptures, so that the students could explain the why, what and their inner feelings. If this was done it should, I feel, be better included in the paper.I was also a little confused with the pre and post questionnaires- there appears to be no matching of expected and actual outcomes, which again I feel might have improved the results.Saying all that, I did enjoy reading the paper and its innovation. One big question, how do we take these findings into the curriculum - having such a course as a stand-alone activity I am not sure gives it the value it deserves. Should the authors follow this group of students through the early part of their course? Its good that the paper creates more questions than answers.

Reviewer Expertise:

NA

No decision status is available

# Reviewer report: Reviewer response for version 1

**DOI:** 10.21956/mep.19646.r29290

**Published:** 2018-09-10

**Authors:** P Ravi Shankar

**Affiliations:** 1 American International Medical University

This review has been migrated. The reviewer awarded 4 stars out of 5 This is an interesting paper describing a creative workshop conducted at Belfast University, Northern Ireland. The workshop was successful in helping collaboration between the participants and increasing their understanding of the links between medicine and art. The photographs of the creations by various groups are interesting.I would be interested in knowing more details about the workshop. There were 28 participants in the workshop. Was 28 kept as the upper limit for the workshop? Did the cadaveric dissection laboratory provide sufficient space for the workshop? How many hours a day did the participants spend working on their projects? How many groups were there? How were the groups selected? Who were the faculty members? How were they selected? Did faculty participants also create artworks?I am not completely sure about the last sentence in the Conclusion section. The authors encountered rich creativity and learning among the participants but a causal relationship may be more difficult to prove. A similar result may have been observed in a different setting outside the cadaveric laboratory. The workshop opened up pathways for connecting between students and faculty members in various professions and areas of study. The article would be of interest to all medical educators.

Reviewer Expertise:

NA

No decision status is available
